# Sustained Release of Minor-Groove-Binding Antibiotic Netropsin from Calcium-Coated Groove-Rich DNA Particles

**DOI:** 10.3390/pharmaceutics11080387

**Published:** 2019-08-02

**Authors:** Hyunsu Jeon, Hyangsu Nam, Jong Bum Lee

**Affiliations:** Department of Chemical Engineering, University of Seoul, 163 Seoulsiripdaero, Dongdaemungu, Seoul 02504, Korea

**Keywords:** DNA particle (DNAp), netropsin, minor groove binder, calcium, rolling circle amplification (RCA)

## Abstract

Control of the release properties of drugs has been considered a key factor in the development of drug delivery systems (DDSs). However, drug delivery has limitations including cytotoxicity, low loading efficiency, and burst release. To overcome these challenges, nano or micro-particles have been suggested as carrier systems to deliver chemical drugs. Herein, nano-sized DNA particles (DNAp) were manufactured to deliver netropsin, which is known to bind to DNA minor grooves. The rationally designed particles with exposed rich minor grooves were prepared by DNAp synthesis via rolling circle amplification (RCA). DNAp could load large quantities of netropsin in its minor grooves. An analytical method was also developed for the quantification of netropsin binding to DNAp by UV–visible spectrometry. Moreover, controlled release of netropsin was achieved by forming a layer of Ca^2+^ on the DNAp (CaDNAp). As a proof of concept, the sustained release of netropsin by CaDNAp highlights the potential of the DNAp-based delivery approach.

## 1. Introduction

Deoxyribonucleic acid (DNA) is a well-known biopolymer that transfers genetic information as part of the central dogma of molecular biology [1,2]. Recent developments in DNA nanotechnology have included its applications in the field of materials science [3,4,5,6]. Researchers have utilized DNA base-pairing to control the shape and size of particles and nanostructures [7]. In this respect, DNA engineering is extensively used to develop novel functional biomaterials. For example, a variety of DNA-based nanomaterials have been developed, including branched DNA [8,9,10], DNA tiles [11,12], DNA polygons [13,14], and DNA origami [15,16,17]. To generate a more diverse range of DNA structures, rolling circle amplification (RCA) has been used to amplify tandem repeat DNA [18,19,20]. In previous studies, RCA-based DNA products, including spherical DNA particles (DNAp) [21,22,23,24] and DNA hydrogels [25,26], were prepared with highly entangled and packed DNA strands.

A major strength of DNA-based materials is the capability for loading DNA-binding molecules of interest in the DNA double helix [27,28]. The hydrophobic portion of the helix structure can carry certain chemicals containing hydrophobic moieties [28,29,30,31,32]. Chemicals that bind to DNA can be categorized as intercalator or minor groove binders depending on their binding position on DNA [33]. Intercalators are physically intercalated into the base pair at the center of the DNA double helix, whereas minor groove binders bind by intercalating into the minor groove of the double helix [34,35]. To date, many DNA binders have been reported as specific functional molecules [36,37,38], and they include fluorescence dyes [39], anticancer drugs [40], and antibiotics [41].

Although chemical drugs have strong efficacy, they often produce strong side effects, including cytotoxicity, low loading efficiency, and burst release. Therefore, it is important to release chemical drugs sustainably to increase the duration of their effects. Therefore, DNA-based drug delivery systems combining DNA structures and DNA-binding drugs are promising systems that have the potential to overcome these limitations [42,43,44,45,46]. Herein, a novel approach for the preparation of a DNA-based antibiotics carrier with DNAp as a vehicle for efficient loading of netropsin, a well-known minor-groove-binding antibiotic, was developed [47]. A strategy for quantitative analysis of netropsin by UV–vis was also developed, which confirmed the loading profile of netropsin from netropsin-loaded DNAp (DNAp@Ne). Moreover, by generating a calcium layer on the particle surface [48], long-term netropsin release from Ca^2+^-coated DNAp@Ne was achieved.

## 2. Materials and Methods

### 2.1. Overview of CaDNAp@Ne

**Figure 1:**
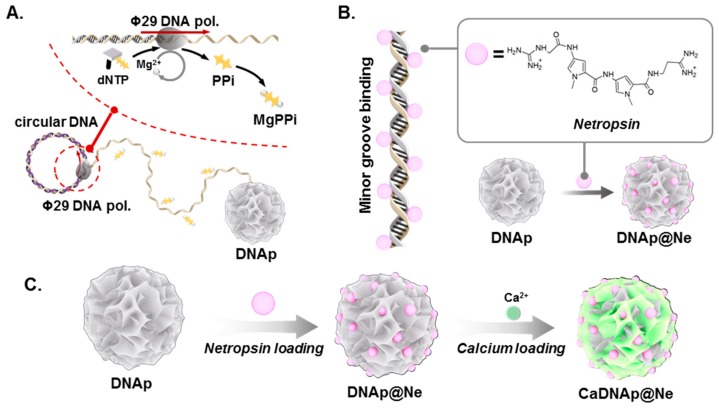
Schematic illustration of CaDNAp@Ne synthesis. (**A**) Scheme for DNA particle (DNAp) synthesis via rolling circle amplification (RCA). (**B**) Scheme of netropsin loading and netropsin-loaded DNAp (DNAp@Ne) synthesis. Because netropsin is a well-known minor groove binder of double-strand DNA, DNAp was used as a supporting matrix for netropsin. (**C**) Scheme for synthesizing DNAp@Ne from netropsin loading to DNAp and CaDNAp@Ne from Ca^2+^ coating of DNAp@Ne.

CaDNAp@Ne was synthesized in three major steps: (i) synthesis of DNAp via RCA, (ii) synthesis of DNAp@Ne via netropsin loading, and (iii) synthesis of CaDNAp@Ne via Ca^2+^ coating of DNAp@Ne, as shown in Figure 1A–C, respectively.

In the first step, DNAp was synthesized by RCA, which promoted DNA polymerization using Ф29 DNA polymerase with circular DNA as a template. Using the circular DNA template, Ф29 DNA polymerase polymerized the elongated strand of DNA. Pyrophosphate (PPi), which is produced during the polymerization process, and Mg^2+^, a cofactor of Ф29 DNA polymerase, were reacted to form magnesium pyrophosphate (Mg_2_PPi), inducing DNAp synthesis with a spherical particle shape (Figure S1) [49,50].

DNAp can achieve specific compound binding via intercalation and minor groove binding. Herein, netropsin, a well-known antibiotic and DNA minor-groove-binding compound, was used as a model drug [47]. To control the release dynamics of netropsin, Ca^2+^ was coated onto DNAp@Ne for surface modification.

### 2.2. Materials and Reagents

DNA oligonucleotides were purchased from Integrated DNA Technologies (IDT), and T4 DNA ligase was purchased from Promega (Madison, WI, USA). Nxgen Ф29 DNA polymerase including 10 × Ф29 polymerase buffer was purchased from Lucigen (Madison, WI, USA). Netropsin and calcium chloride (CaCl_2_) were purchased from Sigma-Aldrich (St. Louis, MO, USA). Dulbecco′s phosphate-buffered saline (DPBS) was purchased from Gibco (Waltham, MA, USA), and a calcium assay kit was purchased from Cayman Chemical (Ann Arbor, MI, USA).

### 2.3. Fabrication of Circular DNA from Linear DNA

Phosphorylated 92-base-long linear DNA and 22-base-long primer DNA at a final concentration of 10 μM were mixed in nuclease-free water. For denaturation and annealing, the mixture was heated to 95 °C for 2 min and cooled slowly to 25 °C for 1 h using a thermal cycler (Bio-Rad). To ligate the nick in the circular DNA, the solution was incubated overnight at room temperature with 0.12 U μL^−1^ of T4 DNA ligase and ligase buffer (30 mM Tris-HCl (pH 7.8), 10 mM MgCl_2_, 10 mM dithiothreitol (DTT), and 1 mM adenosine triphosphate (ATP).

### 2.4. Synthesis of DNAp by RCA

The circular template DNA (final concentration of 1 μM) was incubated with phi29 DNA polymerase (1 U μL^−1^), dNTP mix (2.0 mM for each base), and 2× reaction buffer (100 mM Tris-HCl, 20 mM (NH_4_)_2_SO_4_, 8 mM DTT, and 20 mM MgCl_2_) for 20 h at 30 °C. For washing and purifying DNAp, the reactant was sonicated for 20 min to destroy the microstructural networks connecting DNAp, separating them. The DNAp were subsequently centrifuged at 8000 rpm for 5 min to remove the supernatant, after adding nuclease-free water at twice the initial volume. This washing step was repeated three times, and nuclease-free water was re-filled to recover the initial volume. After washing, the solution was sonicated for 5 min three times. Finally, the concentration of DNAp was measured using a Nanodrop 2000c instrument (Thermo Fisher Scientific, Waltham, MA, USA).

### 2.5. Synthesis of DNAp@Ne

#### 2.5.1. Synthesis of DNAp@Ne via Netropsin Loading

For loading netropsin to DNAp, 100 ng μL^−1^ DNAp was incubated in a 100 μM netropsin aqueous solution at 25 °C overnight. After the reaction, the reactant was centrifuged at 8000 rpm for 5 min to remove the supernatant, after adding nuclease-free water to reach twice the initial volume. This washing step was repeated three times, and nuclease-free water was re-filled to recover the initial volume. After washing, the solution was sonicated for 5 min to prevent aggregation caused by centrifugation.

#### 2.5.2. Stoichiometric Analysis of the Netropsin Loading Process

First, 100 ng μL^−1^ DNAp was incubated in a 100 μM netropsin aqueous solution at 25 °C (DNAp@Ne). As a control group, 100 ng μL^−1^ DNAp was incubated in nuclease-free water at 25 °C (DNAp). After 0, 30, and 60 min, 10 μL each of DNAp and DNAp@Ne were collected, and the supernatants were separated by centrifugation at 8000 rpm for 5 min. The UV–visible light absorbance spectra of the supernatants were analyzed using the Nanodrop 2000c instrument (Thermo Fisher Scientific, Waltham, MA, USA). Measurement of the netropsin loading to DNAp was performed three times under the same experimental conditions, and the average values are reported.

### 2.6. Synthesis of CaDNAp via Coating of Ca^2+^ on DNAp

#### 2.6.1. Synthesis of CaDNAp via Ca^2+^ Coating on DNAp

For coating Ca^2+^ on DNAp, 100 ng μL^−1^ DNAp was incubated in diluted CaCl_2_ solutions (0, 10, and 100 mM) at 25 °C overnight. After the reaction, the reactant was centrifuged at 8000 rpm for 5 min to remove the supernatant, after adding nuclease-free water to twice the initial volume. This washing step was repeated three times, and nuclease-free water was re-filled to recover the initial volume. After washing, the solution was sonicated for 5 min for separation into individual CaDNAp materials. CaDNAp@Ne was synthesized using the same procedure as for DNAp@Ne.

#### 2.6.2. Stoichiometric Analysis of the Ca^2+^ Coating Process

To estimate the stoichiometric Ca^2+^ amount consumed during the coating of DNAp, 100 ng μL^−1^ DNAp was incubated in diluted CaCl_2_ solutions (0, 10, and 100 mM) at 25 °C. The DNAp samples coated with each calcium concentration were named low-CaDNAp (CaCl_2_: 10 mM) and high-CaDNAp (CaCl_2_: 100 mM).

After 0, 0.5, 1, 3, 6, 12, and 24 h, 10 μL each of DNAp and CaDNAp were collected from the solution. The supernatants of each samples were separated by centrifugation at 8000 rpm for 5 min. After separating particles in the solution, the Ca^2+^ concentration in the supernatant was estimated using a calcium assay kit. The samples were processed according to the manufacturer’s instructions provided with the calcium assay kit, and the absorbance at 595 nm was measured using a plate reader. Finally, the Ca^2+^ consumption during coating was evaluated and the absorbance in the supernatant was compared to that of the Ca^2+^ standard. Measurements related to the Ca^2+^ coating of DNAp were performed four times under the same experimental conditions.

#### 2.6.3. Characterization of CaDNAp

For scanning electron microscopy (SEM) analysis, 2 μL of 10 ng μL^−1^ CaDNAp was added to a Si wafer and dried for 3 h. For imaging, the dried sample was inserted into the SEM instrument (Hitachi Fe-SEM SU-70) and analyzed under a 15.0 kV operation voltage.

For transmission electron microscopy (TEM) and energy-dispersive X-ray spectroscopy (EDS) mapping, 2 μL of 10 ng μL^−1^ CaDNAp was added to a lacey TEM grid and dried for 3 h. For TEM imaging, the dried sample was placed in the TEM instrument (JOEL Ltd., Tokyo, Japan, JEM-2100F) operated at 200.0 kV.

### 2.7. Netropsin Stoichiometric Release Analysis from CaDNAp@Ne

Based on the mass of DNAp prior to calcium coating, 5 μg of DNAp@Ne, low-CaDNAp@Ne, and high-CaDNAp@Ne were prepared to generate a netropsin release profile according to the calcium coating degree of the CaDNAp@Ne. After each sample was treated with 50 μL of DPBS, centrifugation at 8000 rpm for 5 min was performed for 0, 3, 6, 12, 24, 48, 72, 96, 120, and 144 h. After centrifugation, 45 μL of the supernatant was collected and re-filled with DPBS. The netropsin concentration in the supernatant was measured using a UV–vis spectrometer, and the netropsin release profile for each sample was determined using an accumulative method. Measurement of the netropsin release profile from CaDNAp@Ne was performed in triplicate under the same experimental conditions, and the average values are reported.

### 2.8. Viability Assay of CaDNAp@Ne

The viability assay for CaDNAp@Ne was measured using a cell counting kit-8 (CCK-8, Dojindo Molecular Technologies, Rockville, MD, USA). HUVEC (Lonza Walkersville Inc., Walkersville, MD, USA) and HepG2 (Korean cell line bank, Seoul, Korea) cells were seeded at a density of 7 × 10^4^ cells/well in a 96-well plate (SPL Life Sciences, Pocheon-si, Korea) and incubated for 24 h. The medium was exchanged with Opti-MEM and incubated for 4 h to promote cell starvation. After 4 h, the cells were treated with CaDNAp@Ne and in control groups for 12 h in serum-free media. After removal of the CaDNAp@Ne-containing serum-free media, the CCK-8 assay was performed based on the manufacturer’s instructions as follows: addition of CCK-8 solution, incubation at 37 °C for 1.5 h, and measurement of the absorbance at 450 nm using a Synergy HT microplate reader (BioTek, Winooski, VT, USA).

## 3. Results

### 3.1. Stoichiometric Analysis of the Netropsin Loading Process

**Figure 2:**
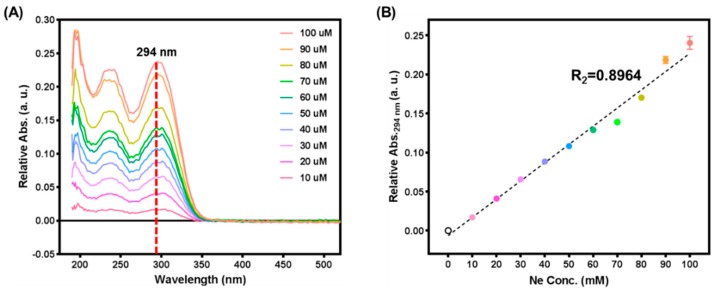
Stoichiometric analysis of netropsin. (**A**) UV–vis spectra of 0 to 100 μM netropsin in aqueous solution. Netropsin showed major peaks at 245 and 294 nm, and 294 nm was selected as it did not overlap with the major peak of DNAp (260 nm). (**B**) Linear regression plot for the netropsin concentration dependence of the absorbance at 294 nm.

To analyze netropsin loading and release profiles for DNAp, measurements were performed to determine the netropsin concentration. Figure 2 shows the absorbance spectrum of a netropsin solution obtained using a UV–vis spectrometer and the standard linear regression plot measured at 294 nm. As shown in Figure 2A, as the concentration of netropsin increased, the absorbance attributed to netropsin increased while maintaining the same spectral envelope. Netropsin showed major absorbance peaks at 240 and 294 nm due to its specific chemical structure. To avoid confusion with the absorbance peak at 260 nm of DNA, 294 nm was chosen as the standard wavelength to generate the standard linear curve for measuring the netropsin concentration (Figure 2B). The absorbance at 294 nm as a function of the netropsin concentration is described in Equation (1):

(1) $$Abs._{294\;nm}=2.334\times 10^{-3}\;C_{Ne}-6.273\;\times 10^{-3}$$

### 3.2. Synthesis of DNAp@Ne via Netropsin Loading to DNAp

**Figure 3:**
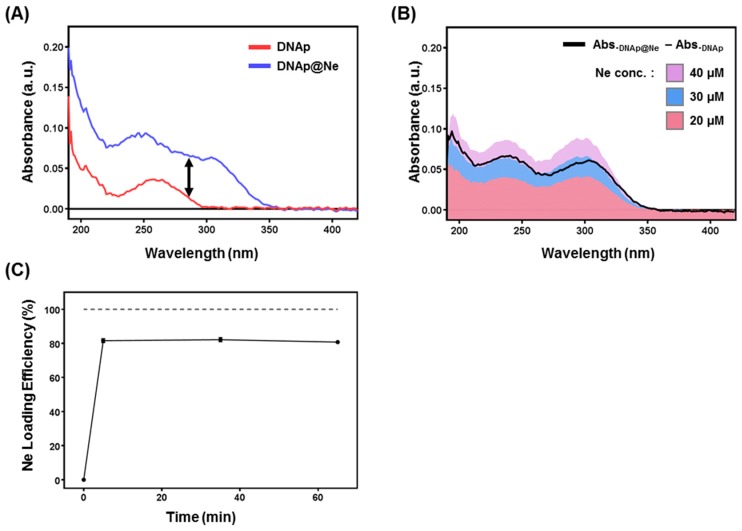
Stoichiometric analysis of netropsin loading on DNAp. (**A**) UV–vis spectra of DNAp and DNAp@Ne. The netropsin treatment time for synthesizing DNAp@Ne was 6 h. DNAp and DNAp@Ne were adjusted to final concentrations of 100 ng μL^−1^ (DNAp: red, DNAp@Ne: blue). (**B**) Stoichiometric analysis of netropsin in DNAp@Ne. The increase in UV–vis absorbance of DNAp@Ne as a function of the absorbance of DNAp (black line) was similar to that of the free netropsin (netropsin 40 μM: purple, 30 μM: blue, 20 μM: red). (**C**) Time-dependent netropsin loading profile on DNAp@Ne where 100 μM of netropsin was added to a solution of 100 ng μL^−1^ of DNAp and measured at various time points.

DNAp@Ne was prepared by loading netropsin to DNAp, and Figure 3A,B shows the quantification of netropsin loaded into DNAp@Ne. By loading netropsin into DNAp, the absorbance spectrum of DNAp increased (Figure 3A). Moreover, the increasing UV–vis absorbance due to netropsin loading showed similar trends as the absorbance spectrum of the free netropsin in aqueous solution (Figure 3B). Based on Figure 3A,B, a method was developed to quantify the netropsin content in DNAp@Ne. The time-dependent loading efficiency of DNAp@Ne was measured and the results are shown in Figure 3C. Netropsin was loaded into DNAp with an efficiency of 80% within 5 min and maintained a stable concentration in the formed DNAp@Ne. In the context of the ratio of drug to carrier for a drug delivery system (DDS), 1 mg of DNAp could incorporate 0.4 mg of netropsin.

### 3.3. Synthesis and Characterization of CaDNAp

**Figure 4:**
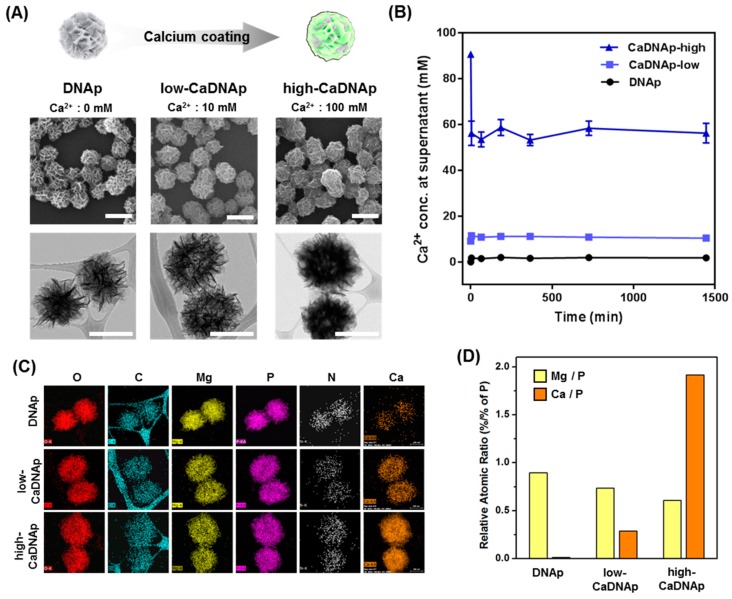
Synthesis and characterization of CaDNAp via Ca^2+^ coating of DNAp. (**A**) Schematic illustration for synthesis of CaDNAp (**top**) and SEM (**middle**) and TEM images (**bottom**) of DNAp, low-CaDNAp, and high-CaDNAp (scale bar: 1 μm). (**B**) Analysis of Ca^2+^ content quantification coated on CaDNAp. (**C**) TEM-EDS mapping images of DNAp (**top row**), low-CaDNAp (**middle row**), and high-CaDNAp (**bottom row**). Each element is indicated above the image. (**D**) The atomic ratios of magnesium and calcium to phosphorus for DNAp, low-CaDNAp, and high-CaDNAp. Atomic percentage data were obtained from TEM-EDS.

CaDNAp was synthesized by coating Ca^2+^ onto DNAp. Previous studies showed that RNAp synthesized using the rolling circle transcription (RCT) technique [51] could be coated with Ca^2+^ by forming an additional layer [48]. Herein, CaDNAp was prepared using a simple dipping method on DNAp. To determine the degree of Ca^2+^ coating, low-concentration (Ca^2+^: 10 mM) and high-concentration (Ca^2+^: 100 mM) conditions were compared, and the particles synthesized by each coating process are referred to as low-CaDNAp and high-CaDNAp, respectively. A schematic illustration (top) and SEM (middle) and TEM (bottom) images are provided in Figure 4A. The SEM images (Figure 4A, middle) show the degree of Ca^2+^ layering on the surface of DNAp. The surface morphology of the DNA was changed to a thicker sponge shape upon coating. Even with an increased concentration of coated Ca^2+^, the original shape and size of the DNAp were retained. The TEM images (Figure 4A, bottom) show that each particle became darker with increasing Ca^2+^ concentration. This was caused by the reduced transmission of electrons due to the increased Ca^2+^ content in the CaDNAp.

To measure the consumption of Ca^2+^ via coating, a calcium assay kit was used to measure the Ca^2+^ concentration in solution. As shown in Figure 4B, CaDNAp synthesis by Ca^2+^ coating occurred within 5 min, and the synthesized CaDNAp stably retained its form bound to calcium for up to 24 h.

In terms of the consumption of calcium, the bonding of Ca^2+^ to the surface of CaDNAp was estimated via EDS mapping (Figure 4C). The DNA particles can be detected by imaging the carbon, nitrogen, oxygen, and phosphorus that constitute DNA and the magnesium that constitutes Mg_2_PPi. Herein, for CaDNAp, a calcium-specific signal was observed at the same site as DNAp, confirming that the calcium consumed (shown in Figure 4B) was coated on the surface of DNAp to form CaDNAp. Moreover, the Mg-to-P and Ca-to-P ratios were compared for the DNAp, low-CaDNAp, and high-CaDNAp samples based on the atomic contents measured by EDS mapping (Figure 4D). Similar to a previous study of CaRNAp [48], it was confirmed that as the degree of Ca^2+^ coating increased, the Ca-to-P ratio increased and that of Mg to P decreased.

### 3.4. Evaluation of the Antibiotic Effects of CaDNAp@Ne

**Figure 5:**
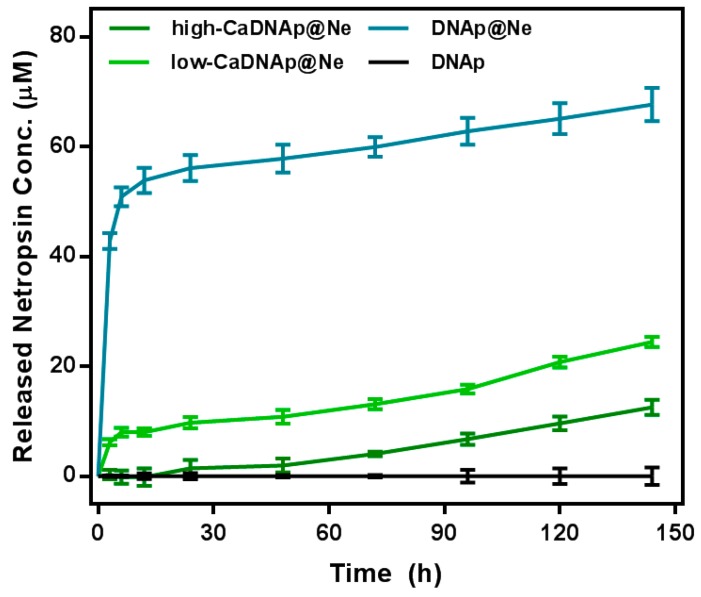
Netropsin release efficiency of DNAp@Ne and CaDNAp@Ne. A cumulative graph of the three samples produced under the same conditions is shown (*n* = 3). For each time point, the sample supernatant was measured using a UV–vis spectrometer (DNAp: black, DNAp@Ne: blue, low-CaDNAp@Ne: light green, high-CaDNAp@Ne: green).

Because netropsin in DNAp@Ne is physically bound to the minor groove of the double helix DNA structure, it was expected to be released into solution over time. Here, Ca^2+^ coating was used to control the netropsin release profile for long-term and stable release. The release of netropsin was caused by the concentration imbalance of the solution and DNAp surface. Thus, the equilibration time of this imbalance could be slowed using the physical barrier created by the Ca^2+^ coating on the surface of DNAp@Ne. As shown in Figure 5, the release rate of netropsin was slowed in both Ca^2+^-coated samples (low-CaDNAp@Ne and high-CaDNAp@Ne). The netropsin loading rate at the initial stage was similar, while the netropsin release rate was low for low-CaDNAp@Ne and high-CaDNAp@Ne, suggesting that release over a long time period is possible.

## 4. Discussions

Herein, a netropsin delivery method was developed using DNAp as a template. For the synthesis of DNAp, RCA was applied to circular DNA and DNAp@Ne was synthesized by successfully loading netropsin into the formed DNAp.

Netropsin stoichiometric analysis using UV–vis spectroscopy was also newly developed. Because the absorbance peak at 294 nm was formed due to the specific molecular structure of netropsin and could be distinguished from the absorbance peak at 260 nm arising from DNA, quantification of the netropsin concentration in solution was achieved using a standard curve generated via UV–vis spectroscopy.

Therefore, it was possible to determine the binding ratio of DNA contained in DNAp@Ne to netropsin. The netropsin–DNA binding ratio was calculated to be 80 μM netropsin per 100 ng μL^−1^ of DNA, yielding a loading rate of 1.05 mol netropsin per 1 mol DNA base pairs.

Furthermore, Ca^2+^ coating of DNAp@Ne was performed to achieve sustained release of netropsin. Similar to a previous study of RNAp [48], coating DNAp with Ca^2+^ formed a new layer on the surface. Moreover, this newly formed layer was composed of calcium, and various stoichiometries were used for the coating and shown by atomic analysis through EDS mapping.

For the CaDNAp@Ne, because Ca^2+^ coating proceeded after netropsin loading, it was expected that netropsin release from the DNAp matrix would be physically hindered. As expected, the release rate of netropsin was controlled by forming a layer on the surface of the DNAp@Ne with Ca^2+^, and sustained release from the particles was confirmed. The application of calcium to DNA-based template particles to regulate drug release profiles is highly promising because it is possible to design a DDS entirely composed of biocompatible materials. As demonstrated in Figure S2, no specific cytotoxicity against the HUVEC cell line (as a normal cell test group) was observed, and the HepG2 cell line, used as a cancer cell test line, for all sample groups showed no adverse effects upon exposure to DNAp.

In conclusion, due to the abundant binding sites on DNAp, DNA minor groove binding of netropsin was successfully achieved and sustained release could be systemically adjusted, indicating the potential of the prepared DNA particles as a general groove-binding drug carrier. Numerous groove-binding drugs have been developed, including antitumor compounds (e.g., doxorubicin [32,43,46] and daunorubicin [52]) and antibiotics (e.g., plicamycin and chromomycin A3 [53]). In future, these compounds will be applied to the system developed herein to generate an optimal DDS for each therapeutic compound.

## Supplementary Materials

The following are available online at https://www.mdpi.com/1999-4923/11/8/387/s1, Figure S1: Size distribution of the DNAp-based netropsin release systems. (DNAp: black, DNAp@Ne: blue, low-CaDNAp@Ne: light green, high-CaDNAp@Ne: green); Figure S2: Cell viability when exposed to DNAp-based netropsin release systems. The viability of the CaDNAp@Ne and control groups was tested using the normal endothelial cell line HUVEC and the cancer cell line HepG2.
