# Supplementary material for: Sustained Release of Minor-Groove-Binding Antibiotic Netropsin from Calcium-Coated Groove-Rich DNA Particles
Source: Pharmaceutics. 2019 Aug 2;11(8):387. doi: 10.3390/pharmaceutics11080387 (PMC6724015; doi:10.3390/pharmaceutics11080387)
Supplement: Supplementary file 1 [file pharmaceutics-11-00387-s001.pdf]

pharmaceutics

Article

Sustained Release of Minor-Groove-Binding Antibiotic Netropsin from Calcium-Coated Groove-Rich DNA Particles

Hyunsu Jeon†, Hyangsu Nam† and Jong Bum Lee*†

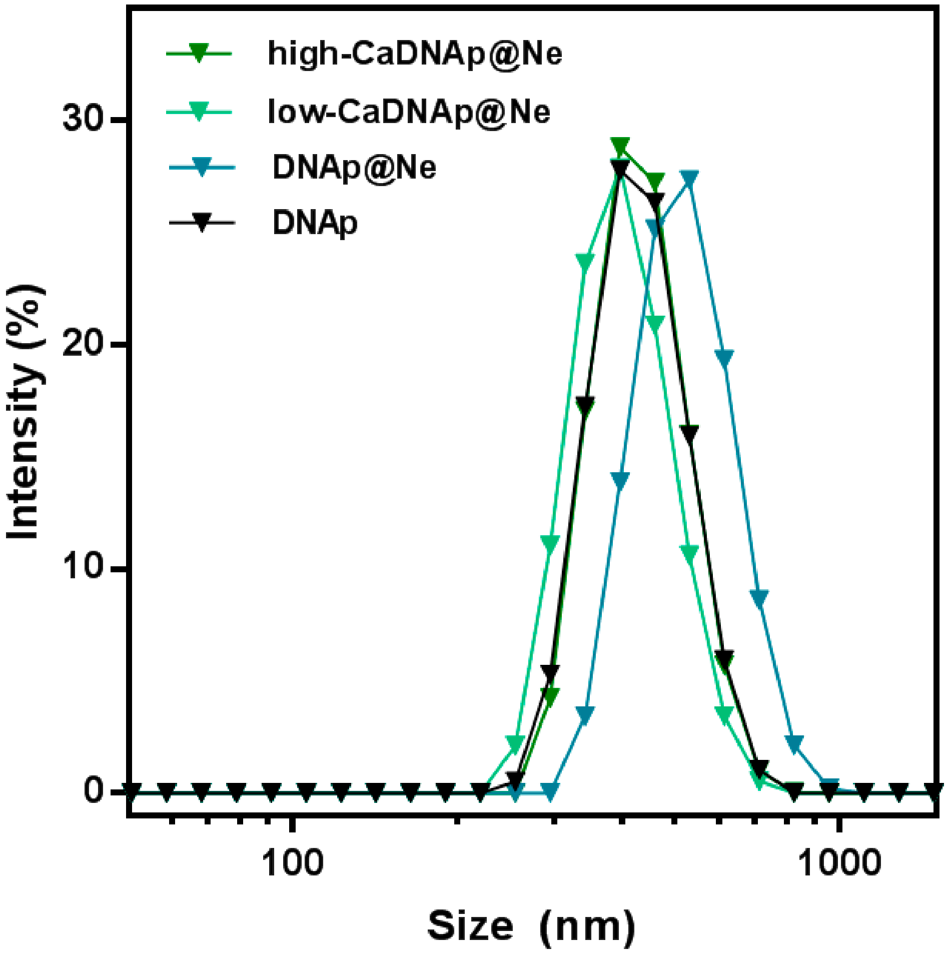

Figure S1. Size distribution of the DNAp-based netropsin release systems. (DNAp: black, DNAp@Ne: blue, low-CaDNAp@Ne: light green, high-CaDNAp@Ne: green).

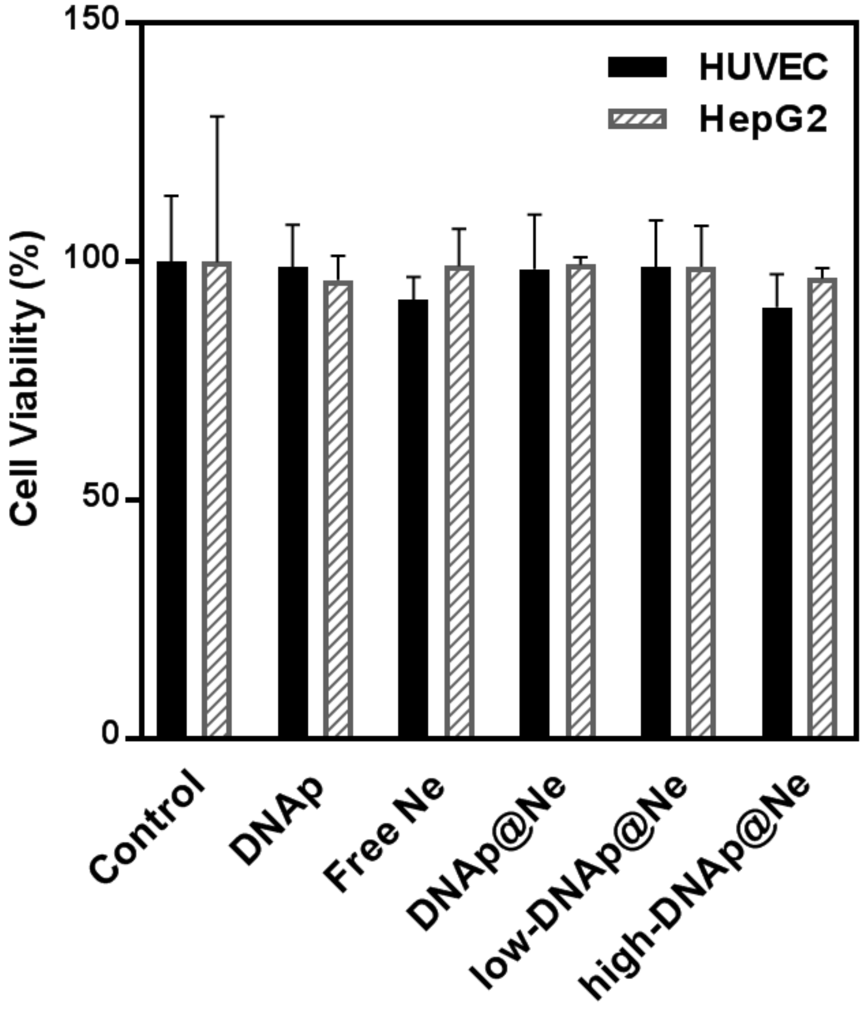

Figure S2. Cell viability when exposed to DNAp-based netropsin release systems. The viability of the CaDNAp@Ne and control groups was tested using the normal endothelial cell line HUVEC and the cancer cell line HepG2.

© 2019 by the authors. Submitted for possible open access publication under the terms and conditions of the Creative Commons Attribution (CC BY) license (http://creativecommons.org/licenses/by/4.0/).
